# Similar efficacy of ibrutinib arms across ALPINE and ELEVATE-RR trials in relapsed/refractory chronic lymphocytic leukemia: a matching-adjusted indirect comparison

**DOI:** 10.1038/s41408-024-01044-4

**Published:** 2024-05-02

**Authors:** Mazyar Shadman, Alessandra Tedeschi, Leyla Mohseninejad, Keri Yang, Nicole Lamanna, Sheng Xu, Aileen Cohen, Swetha Challagulla, Mei Xue, Rhys Williams, Susan M. O’Brien, Jennifer R. Brown, Constantine Tam

**Affiliations:** 1 Fred Hutchinson Cancer Research Center and University of Washington, Seattle, WA USA; 2 ASST Grande Ospedale Metropolitano Niguarda, Milan, Italy; 3 BeiGene, Netherlands B.V., Schiphol, The Netherlands; 4 BeiGene USA Inc, San Mateo, CA USA; 5 Herbert Irving Comprehensive Cancer Center, Columbia University, New York, NY USA; 6 BeiGene (Beijing) Co. Ltd., Beijing, China; 7 Chao Family Comprehensive Cancer Center, University of California, Irvine, CA USA; 8 Dana–Farber Cancer Institute, Boston, MA USA; 9 The Alfred Hospital and Monash University, Melbourne, VIC Australia

**Keywords:** Health care, Therapeutics

Dear Editor,

Two different randomized controlled trials (RCTs) have compared, head-to-head, the efficacy and safety of Bruton tyrosine kinase inhibitors (BTKis) in chronic lymphocytic leukemia (CLL); in both these studies, the first-generation BTKi ibrutinib was used as the comparator arm. ELEVATE-RR (NCT02477696), a multicenter, randomized, open-label, noninferiority phase 3 trial, compared acalabrutinib vs. ibrutinib in patients with previously treated, high-risk [presence of del(17p) and/or del(11q)] CLL [1]. In this study, acalabrutinib met its primary endpoint of progression-free survival (PFS) noninferiority (hazard ratio [HR]:1.0; 95% confidence interval [CI], 0.79–1.27) with a median PFS of 38.4 months in both arms. Acalabrutinib demonstrated improved tolerability with fewer cardiovascular adverse events (AEs) vs. ibrutinib.

ALPINE (NCT03734016) was a global, randomized, open-label phase 3 trial designed to assess the superiority of zanubrutinib over ibrutinib in patients with relapsed/refractory (R/R) CLL or small lymphocytic lymphoma [2–4]. In the ALPINE intent-to-treat population, zanubrutinib demonstrated superior PFS compared with ibrutinib when assessed by either an independent review committee (IRC) or by the investigator (INV) [2]. In high-risk patients with del(17p)/*TP53* mutation, as well as across other major subgroups, PFS favored zanubrutinib. Furthermore, zanubrutinib had an improved safety profile compared with ibrutinib with a lower rate of treatment discontinuation and fewer cardiac disorder events, including fewer deaths.

Comparison of ibrutinib arms across separate trials can be made using matching-adjusted indirect comparison (MAIC) methodology, where individual patient-level data (IPD) from one trial are combined with published aggregate data from another trial, followed by propensity score weighting. Baseline characteristics of patients with IPD are weighted, and IPD are reanalyzed to match outcome definitions in the aggregate data [5]. A recent indirect comparison of the ibrutinib arms across the ALPINE, ELEVATE-RR, and RESONATE (ibrutinib vs. ofatumumab) trials using MAIC methodology implied that ibrutinib underperformed in ALPINE [6]. The analysis matched key patient baseline characteristics including age ≥75 years, bulky disease, prior treatments, β_2_-microglobulin, and del(11q) or del(17p) status but omitted other characteristics critical for appropriate cross-trial comparisons, such as sex, *TP53* and immunoglobulin heavy chain variable (IGHV) mutation status, complex karyotype, and Binet stage.

The present study compared the efficacy of the ibrutinib arms across the ALPINE and ELEVATE-RR trials using MAIC methodology and a more comprehensive list of matching variables to address the underperformance of ibrutinib within ALPINE reported by Ghia et al. [6]. As there was no common comparator between ALPINE and ELEVATE-RR when comparing the efficacy of the ibrutinib arms, this study used an unanchored MAIC, which was conducted inline with published recommendations [5]. The ALPINE ibrutinib arm IPD (*N* = 325) were filtered to include patients who met the inclusion criteria of ELEVATE-RR (i.e., R/R CLL with del(17p) or del(11q) deletions). The resulting sample (*N* = 123) was re-weighted to align the distribution of relevant effect modifiers (EMs) and prognostic factors (PFs) with published aggregate data for the ibrutinib arm of ELEVATE-RR (*N* = 265) [1, 2]. Weights were determined using propensity scores. The MAIC was designed to adjust for all relevant EMs and PFs, which were identified based on a review of the impact of different subgroups analyzed in previous CLL trials and confirmed with clinical experts. The selected parameters for propensity score weighting in the base case were del(17p), del(11q), *TP53* mutation status, IGHV mutation status, serum β2-microglobulin, number of prior therapies, and Binet stage. Re-weighted IPD were used to calculate adjusted efficacy outcomes in ALPINE. Weighted HRs were estimated to compare PFS-IRC, PFS-INV, and overall survival (OS) between the ibrutinib arms in ALPINE and ELEVATE-RR. Pseudo IPD of time to event outcomes for the ibrutinib arm of ELEVATE-RR were reconstructed from Kaplan-Meier curves reported in the ELEVATE-RR publication using the algorithm by Guyot et al. [7]. HRs of time to event outcomes were estimated from a weighted Cox model (i.e., comparing weighted ibrutinib ALPINE data against the pseudo IPD of ibrutinib in ELEVATE-RR). Nominal *p* values were reported for descriptive purposes.

Sensitivity analyses were performed to assess the robustness of the base case results. In the first sensitivity analysis, additional EMs and PFs, including age, sex, complex karyotype, bulky disease, and Eastern Cooperative Oncology Group Performance Status were adjusted. In a second sensitivity analysis, ALPINE PFS and OS were adjusted for COVID-19 impact, as ALPINE was conducted during the COVID-19 period and ELEVATE-RR follow-up data (included in this analysis) were mostly collected before the COVID pandemic. This was achieved by censoring the patients who died due to COVID-19 at the most recent disease assessment prior to death or at the death due to COVID-19.

Baseline characteristics of the populations before matching and a comprehensive summary of EMs and PFs adjusted in the base case and the sensitivity analyses are summarized in Table 1A. Matching the two populations reduced the effective sample size (ESS) from 123 to 63 in the base case analysis.

**Table 1:** Baseline characteristics before matching and after adjustment for the ibrutinib arms of ALPINE and ELEVATE-RR (A) and PFS-IRC, PFS-INV, and OS (B) in the base case and sensitivity analyses.

**A. Baseline characteristics of ibrutinib arms in ALPINE and ELEVATE-RR before matching, and adjustment for EMs and PFs in base case and sensitivity analyses**								
**Population characteristics**	**High-risk Ibrutinib arm ALPINE trial (*N*** = 123)	**Ibrutinib arm ELEVATE-RR trial (*N*** = 265)	**M1 (base case) ESS** **=** **63**	**M2 ESS** **=** **64**	**M3 ESS** **=** **55**	**M4 ESS** **=** **25**	**M5 ESS** **=** **64**	**M6 ESS** **=** **81**
Age ≥75 (vs. <75), %	22.8	16.2			✓	✓		
Sex (male), %	70.7	73.2			✓	✓		
Mutated IGHV, %	17.4	10.6	✓	✓	✓	✓	✓	✓
Del(17p), %	40.7	45.3	✓	✓	✓	✓	✓	✓
Del(11q), %	71.5	66.1	✓	✓	✓	✓		
Mutated *TP53*, %	20.3	42.3	✓	✓	✓	✓	✓	
Complex karyotype (≥3 abnormalities), %	46.5	47.2				✓		
β2-microglobulin (>3.5 mg/l), %	69.2	80.8	✓	✓	✓	✓	✓	✓
Number of prior therapies ≥4, %	8.1	10.6	✓		✓	✓	✓	✓
Bulky disease (LDi in cm) ≥5, %	50.4	51.3			✓	✓		
ECOG PS 2, %	3.3	8.3			✓	✓		
Binet stage A and B (CLL only), %	66.1	54.2	✓	✓	✓	✓	✓	✓

**B. Base case and sensitivity analyses adjusting for COVID-19 impact**					
**Model**	**Adjustment for COVID?**	**Ibrutinib ESS**	**PFS-INV HR**^**a**^ **(95% CI);** ***p*** **value**	**PFS-IRC HR**^**a**^ **(95% CI);** ***p*** **value**	**OS HR**^**a**^ **(95% CI);** ***p*** **value**
M1 (Base-case)	No	63	1.18 (0.75–1.86); *p* = 0.4827	0.80 (0.49–1.28); *p* = 0.3485	0.91 (0.50–1.65); *p* = 0.7539
	Yes	63	1.11 (0.69–1.77), *p* = 0.6710	0.74 (0.45–1.22), *p* = 0.2362	0.73 (0.37–1.43), *p* = 0.3567
M2	No	64	1.15 (0.73–1.82); *p* = 0.5438	0.78 (0.48–1.26); *p* = 0.3080	0.89 (0.49–1.62); *p* = 0.7110
	Yes	64	1.08 (0.67–1.74), *p* = 0.7459	0.72 (0.44–1.19), *p* = 0.2025	0.71 (0.36–1.40), *p* = 0.3260
M3	No	55	1.05 (0.63–1.75), *p* = 0.8573	0.71 (0.41–1.22), *p* = 0.2141	0.74 (0.36–1.53), *p* = 0.4212
	Yes	55	1.00 (0.59–1.70), *p* = 0.9899	0.67 (0.38–1.18), *p* = 0.1679	0.63 (0.28–1.42), *p* = 0.2684
M4	No	25	1.08 (0.54–2.14); *p* = 0.8309	0.96 (0.51–1.82); *p* = 0.9045	0.77 (0.29–2.07); *p* = 0.6055
	Yes	25	1.00 (0.49–2.05), *p* = 0.9990	0.90 (0.46–1.76), *p* = 0.7631	0.63 (0.20–1.95), *p* = 0.4204
M5	No	64	1.20 (0.77–1.90); *p* = 0.4217	0.82 (0.51–1.32); *p* = 0.4135	0.97 (0.53–1.76); *p* = 0.9141
	Yes	64	1.13 (0.71–1.81), *p* = 0.5996	0.76 (0.47–1.25), *p* = 0.2863	0.78 (0.40–1.53), *p* = 0.4752
M6	No	81	1.31 (0.86–2.00); *p* = 0.2021	0.83 (0.53–1.29); *p* = 0.3990	1.07 (0.61–1.87); *p* = 0.8146
	Yes	81	1.24 (0.80–1.90), *p* = 0.3379	0.76 (0.48–1.21), *p* = 0.2540	0.87 (0.47–1.62), *p* = 0.6577

*CI* confidence interval, *CLL* chronic lymphocytic leukemia, *COVID-19* coronavirus disease-19, *ECOG PS* Eastern Cooperative Oncology Group Performance Status, *EM* effect modifier, *ESS* effective sample size, *HR* hazard ratio, *IGHV* immunoglobulin heavy chain variable, *INV* investigator, *IRC* independent review committee, *LDi* longest diameter, *OS* overall survival, *PF* prognostic factor, *PFS* progression-free survival.

^a^HR presented as ibrutinib ALPINE vs. ibrutinib ELEVATE-RR.

**Fig. 1:**
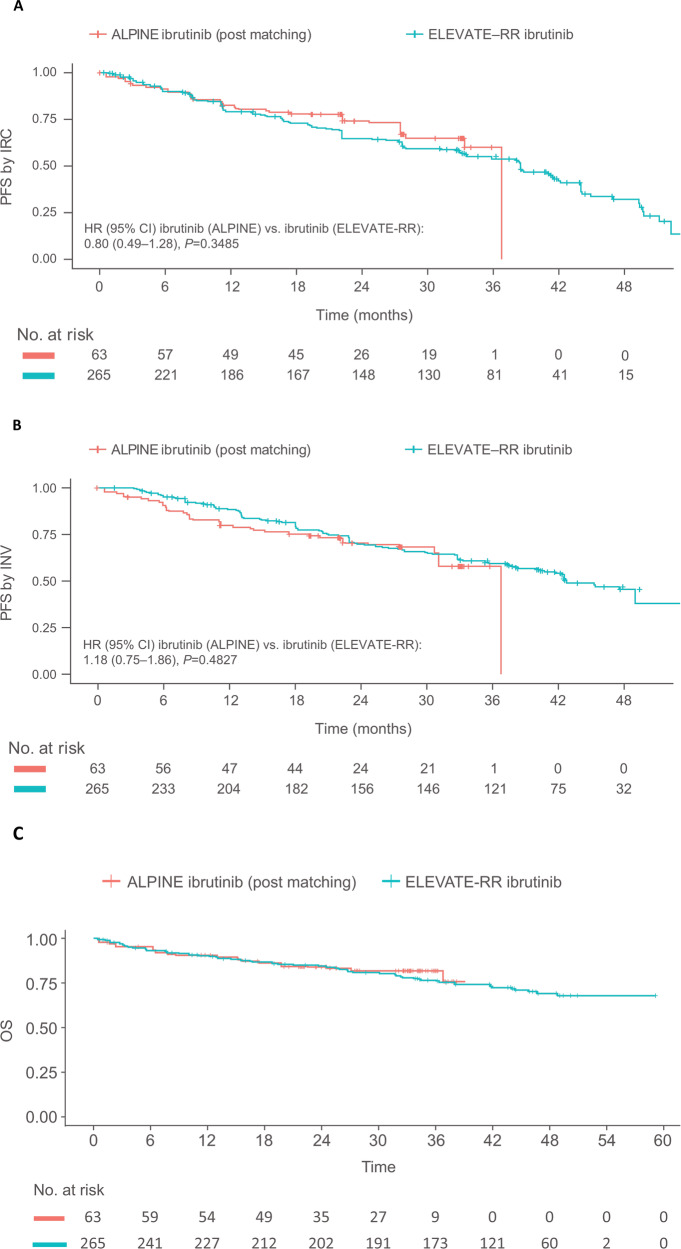
Survival outcomes. **A** PFS-IRC. **B** PFS-INV. **C** OS. ^a^Given the availability of both IRC- and INV-assessed data. CI confidence interval, CLL chronic lymphocytic leukemia, COVID-19 coronavirus disease-19, EM effect modifier, HR hazard ratio, INV investigator, IPD individual patient-level data, IRC independent review committee, MAIC matching-adjusted indirect comparison, ORR overall response rate, OS overall survival, PF prognostic factor, PFS progression-free survival, RCT randomized clinical trial, R/R relapsed refractory, SLL small lymphocytic lymphoma.

The base case PFS-IRC, PFS-INV, and OS for the ibrutinib arms of ALPINE and ELEVATE-RR are shown in Fig. 1 and Table 1B. After matching (median follow-up, 28.4 months), no statistically significant differences were observed in PFS-IRC (HR [95% CI] = 0.80 [0.49–1.28], *p* = 0.3485), PFS-INV (HR [95% CI] = 1.18 [0.75–1.86], *p* = 0.4827), or OS (HR = 0.91 [0.50–1.65], *p* = 0.7539) between the ibrutinib arms of ALPINE and ELEVATE-RR. Findings from the sensitivity analyses for additional EMs and PFs and the COVID-19 adjustment were consistent with those observed for the base case (Table 1B).

While no significant differences were observed between the efficacy outcomes in the ibrutinib arms of ALPINE and ELEVATE-RR, ibrutinib in ALPINE showed numerical “overperformance” compared to ELEVATE-RR with regards to PFS-IRC and OS. This trend could be observed for the base case and sensitivity analyses, where the HRs of PFS-IRC and OS for the ibrutinib arms of ALPINE vs. ELEVATE-RR were always below 1. These observations highlight the importance of considering both PFS-IRC and PFS-INV for unanchored MAICs, where available, as the conclusions may change when using different PFS measurements. However, given that both ALPINE and ELEVATE-RR were open-label trials, PFS-IRC is a preferred endpoint [8, 9].

Findings from the present study contrast with the results of the previous MAIC [6]. The MAIC results from Ghia et al. showed that PFS and overall response rate outcomes for ibrutinib were consistent between RESONATE and ELEVATE-RR but ibrutinib “underperformed” in ALPINE. Findings here demonstrate an equivalence. The disparate findings between the present and previous study may be attributed to differences in the EMs and PFs adjusted for in the MAIC analyses. Several important patient characteristics such as sex, IGHV mutation status, *TP53* mutation status, complex karyotype, and Binet stage were not considered in the Ghia study. Presence of complex karyotype, advanced Binet stage, unmutated IGHV, del(11q), and *TP53* abnormalities are high-risk markers for CLL [10]. Failure to appropriately identify and select EMs and PFs in MAICs may result in biased or uncertain effect estimates, impacting the validity of the analysis [11].

Indirect treatment comparisons such as MAICs provide useful information on the comparative efficacy of treatments evaluated in separate trials, potentially filling evidence gaps for health technology assessments [5, 12]. However, due to limitations (modeling assumptions and cross-trial differences in baseline characteristics) and confounding associated with these methodologies, MAIC analyses cannot replace the gold standard of RCTs, should be interpreted with caution, and be viewed as observational and hypothesis-generating [5, 13].

Like any other MAIC, this study had some limitations. Notably, the ESS of the ibrutinib arm in ALPINE was reduced to 63 after filtering out the non-high-risk patients and conducting the matching and adjustment. The study was by nature limited to the high-risk ALPINE population, which reduced the starting sample size. The ESS was further decreased as all important baseline characteristics were considered for accurate comparisons. Despite the small ESS, results were consistent across the multiple sensitivity analyses tested.

The present study did not evaluate the efficacy of the ibrutinib arm of RESONATE. Given both ELEVATE-RR and ALPINE are more contemporary trials that compare a next-generation BTKi to ibrutinib, ELEVATE-RR was deemed more suitable for this comparison. We would expect ibrutinib to perform slightly better in RESONATE compared to ALPINE, potentially due to (1) the difference between RESONATE and ALPINE with regards to geographic distribution of patients and (2) ibrutinib was the only BTKi available in clinical trials at the time of RESONATE, with the only alternatives being standard of care chemotherapies, possibly leading to enhanced adherence.

In conclusion, this MAIC used a comprehensive list of matching variables to compare the efficacy of the ibrutinib arms in ALPINE and ELEVATE-RR, showing no significant difference in the performance of ibrutinib across the two trials. Results were robust in all sensitivity analyses. While MAICs provide a basis for hypothesis generation with regards to treatment efficacy across trials, they are not a substitute for head-to-head RCTs, as they cannot balance all observable and unobservable differences at baseline. Consequently, ultimate evidence of relative efficacy must be sought within RCTs.
